# Methyl 4-(4-fluoro­anilino)-1,2,6-tris­(4-fluoro­phen­yl)-1,2,5,6-tetra­hydro­pyri­dine-3-carboxyl­ate

**DOI:** 10.1107/S160053681300370X

**Published:** 2013-02-09

**Authors:** Sumati Anthal, Goutam Brahmachari, Suvankar Das, Rajni Kant, Vivek K. Gupta

**Affiliations:** a Post-Graduate Department of Physics & Electronics, University of Jammu, Jammu Tawi 180 006, India; b Laboratory of Natural Products & Organic Synthesis, Department of Chemistry, Visva-Bharati University, Santiniketan 731 235, West Bengal, India

## Abstract

In the title mol­ecule, C_31_H_24_F_4_N_2_O_2_, the tetra­hydro­pyridine ring adopts a distorted boat conformation. An intra­molecular N—H⋯O hydrogen bond is formed by the amino group and ccarboxyl C=O atom. The crystal structure features weak C—H⋯F and C—H⋯O inter­actions.

## Related literature

For biological activity of functionalized piperidine derivatives, see: Zhou *et al.* (2007 ▶); Misra *et al.* (2009 ▶); Bin *et al.* (2001 ▶); Agrawal & Somani (2009 ▶); Jaen *et al.* (1988 ▶). For general background to functionalized piperidines, see: Kamei *et al.* (2005 ▶). For related structures, see: Sambyal *et al.* (2011 ▶); Brahmachari & Das (2012 ▶); Khan *et al.* (2010 ▶); Anthal *et al.* (2013 ▶). For asymmetry parameters, see: Duax *et al.* (1975 ▶).
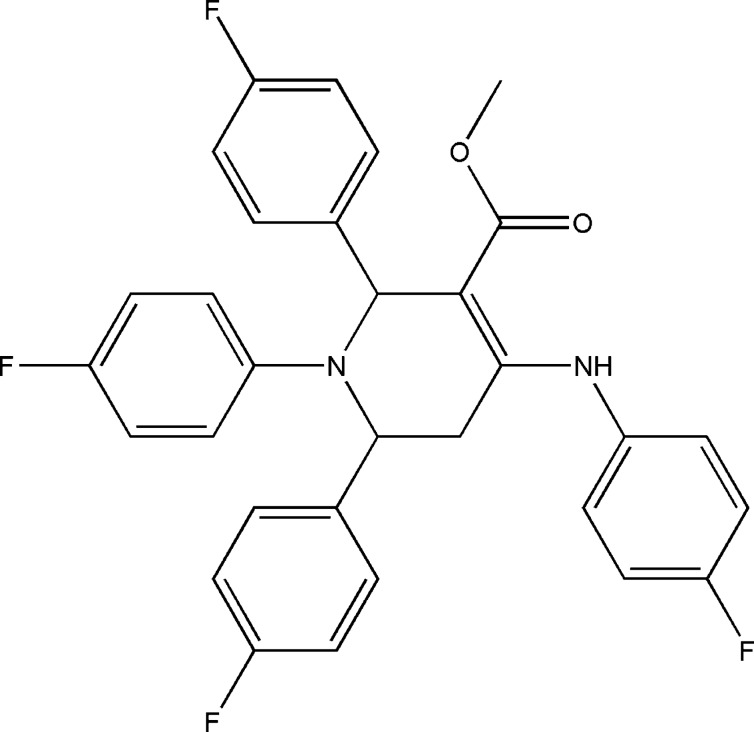


## Experimental

### 

#### Crystal data

C_31_H_24_F_4_N_2_O_2_
*M* *_r_* = 532.52
Triclinic, 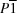

*a* = 9.7990 (2) Å
*b* = 10.7316 (4) Å
*c* = 13.7395 (4) Å
α = 110.797 (3)°
β = 100.338 (2)°
γ = 96.323 (2)°
*V* = 1304.81 (7) Å^3^
*Z* = 2
Mo *K*α radiation
μ = 0.10 mm^−1^
*T* = 293 K
0.30 × 0.20 × 0.20 mm

#### Data collection

Oxford Diffraction Xcalibur Sapphire3 diffractometer
Absorption correction: multi-scan (*CrysAlis RED*; Oxford Diffraction, 2010 ▶) *T*_min_ = 0.899, *T*_max_ = 1.000
42990 measured reflections
5413 independent reflections
3730 reflections with *I* > 2σ(*I*)
*R*_int_ = 0.044

#### Refinement

*R*[*F* ^2^ > 2σ(*F* ^2^)] = 0.046
*wR*(*F* ^2^) = 0.118
*S* = 1.05
5413 reflections
353 parameters
H-atom parameters constrained
Δρ_max_ = 0.17 e Å^−3^
Δρ_min_ = −0.20 e Å^−3^

### 

Data collection: *CrysAlis PRO* (Oxford Diffraction, 2010 ▶); cell refinement: *CrysAlis PRO*; data reduction: *CrysAlis PRO*; program(s) used to solve structure: *SHELXS97* (Sheldrick, 2008 ▶); program(s) used to refine structure: *SHELXL97* (Sheldrick, 2008 ▶); molecular graphics: *ORTEP-3 for Windows* (Farrugia, 2012 ▶); software used to prepare material for publication: *PLATON* (Spek, 2009 ▶).

## Supplementary Material

Crystal structure: contains datablock(s) I, global. DOI: 10.1107/S160053681300370X/gk2551sup1.cif

Structure factors: contains datablock(s) I. DOI: 10.1107/S160053681300370X/gk2551Isup2.hkl

Supplementary material file. DOI: 10.1107/S160053681300370X/gk2551Isup3.cml

Additional supplementary materials:  crystallographic information; 3D view; checkCIF report

## Figures and Tables

**Table 1:** Hydrogen-bond geometry (Å, °)

*D*—H⋯*A*	*D*—H	H⋯*A*	*D*⋯*A*	*D*—H⋯*A*
N9—H9⋯O1	0.86	2.05	2.695 (2)	131
C20—H20⋯F2^i^	0.93	2.54	3.384 (2)	152
C32—H32⋯O1^ii^	0.93	2.47	3.311 (3)	151

Symmetry codes: (i) 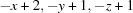; (ii) 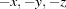.

**Fig. 1.**
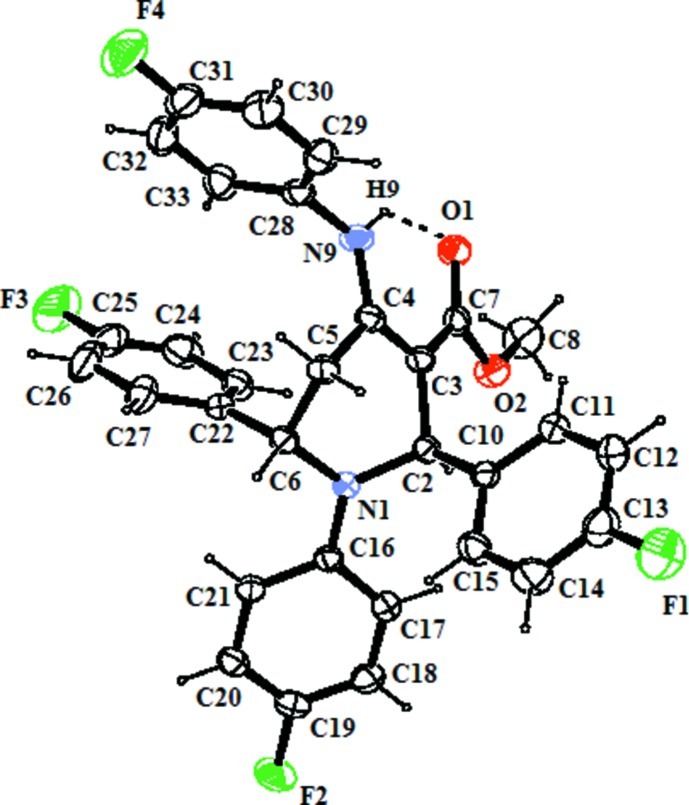
ORTEP view of the title molecule with the atom-labeling scheme. The displacement ellipsoids are drawn at the 40% probability level. H atoms are shown as small spheres of arbitrary radii.

**Fig. 2.**
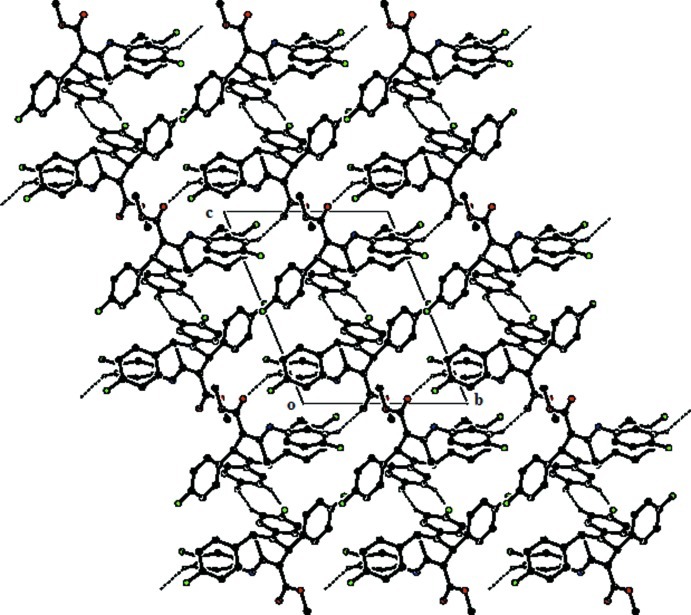
The packing arrangement of molecules viewed down the a axis. Short contacts are shown with dashed lines.

## supplementary crystallographic information

### Comment

Functionalized piperidines, very particularly 1,4-disubstituted piperidine scaffolds, are found to be useful in designing a variety of medicinal entities exhibiting a broad spectrum of pharmacological activities that include antibacterial (Zhou *et al.*, 2007), antimalarial (Misra *et al.*, 2009), anti-hypertensive, anticonvulsant, anti-inflammatory (Bin *et al.*, 2001), and enzyme inhibitory activity (Agrawal & Somani, 2009; Jaen *et al.*, 1988). Moreover, a large number of compounds bearing piperidine scaffold have already entered into preclinical and clinical trials over the last few years (Kamei *et al.*, 2005). Hence, investigation of the structural features of biologically relevant piperidine derivatives is demanding. In continuation of our structural studies of densely functionalized piperidines (Sambyal *et al.*, 2011; Brahmachari & Das, 2012) we present here the crystal structure of the title compound. The molecular structure of the title compound is illustrated in Fig.1. The bond lengths and angles of the title compound are normal and correspond to those observed in related structures (Khan *et al.*, 2010; Anthal *et al.*, 2013). In the title molecule, tetrahydropyridine ring adopts a distorted boat conformation with asymmetry parameters [ΔC_s_(C2)=10.10] and [ΔC_s_(C3-C4)=15.48] (Duax *et al.*, 1975). In the crystal, an intramolecular hydrogen bond N9-H9···O1 is found. This intramolecular interaction leads to the formation of a pseudo-six membered ring comprising atoms O1, C7, C3, C4, N9 and H9. The molecular structure is stablized by N—H···O intramolecular interaction and crystal packing is stablized by C—H···F and C—H···O intermolecular interactions (Table 1). Molecules are linked *via* C—H···F and C—H···O hydrogen bonds to form chains along [010](Fig. 2).

### Experimental

An oven-dried screw cap reaction tube was charged with a magnetic stir bar, 4-fluoroaniline (2 mmol), methyl acetoacetate (1 mmol) and Bi(NO_3_)_3_.5H_2_O (10 mol%) in 4 ml ethanol; the mixture was stirred at room temperature for 20 min, and then 4-fluorobenzaldehyde (2 mmol) was added to the reaction mixture and stirring was continued up to 12 h to complete the reaction (monitored by TLC). On completion of the reaction, a thick white precipitate was obtained. The solid residue was filtered off and washed with cold ethanol–water. The solid mass was dissolved in hot ethyl acetate–ethanol mixture and filtered off when bismuth salt separated out; the filtrate on standing afforded white crystals of the title compound, characterized by elemental analyses and spectral studies including FT—IR, ^1^H-NMR, and ^13^C-NMR. For X-ray study, single crystals were prepared by further recrystallization by slow evaporation from ethanol-ethyl acetate-water solution. Methyl 1,2,6-tris(4-fluorophenyl)-4-((4-fluorophenyl)amino)-1,2,5,6- tetrahydropyridine-3-carboxylate : white crystals; mp 452–454 K. Anal. Calcd for C_31_H_24_F_4_N_2_O_2_: C 69.92, H 4.54, N 5.26; found: C 69.95, H 4.52, N 5.28.

### Refinement

All H atoms were positioned geometrically and were treated as riding on their parent C/N atoms, with C—H distances of 0.93–0.98 Å and N—H distance of 0.86 Å and with *U*_iso_(H) = 1.2*U*_eq_(C/N), except for the methyl groups where *U*_iso_(H) = 1.5*U*_eq_(C).

### Crystal data

**Table:**

C_31_H_24_F_4_N_2_O_2_	*Z* = 2
*M**_r_* = 532.52	*F*(000) = 552
Triclinic, *P*1	*D*_x_ = 1.355 Mg m^−^^3^
Hall symbol: -P 1	Mo *K*α radiation, λ = 0.71073 Å
*a* = 9.7990 (2) Å	Cell parameters from 14371 reflections
*b* = 10.7316 (4) Å	θ = 3.5–29.0°
*c* = 13.7395 (4) Å	µ = 0.10 mm^−^^1^
α = 110.797 (3)°	*T* = 293 K
β = 100.338 (2)°	Block, white
γ = 96.323 (2)°	0.30 × 0.20 × 0.20 mm
*V* = 1304.81 (7) Å^3^	

### Data collection

**Table:**

Oxford Diffraction Xcalibur Sapphire3 diffractometer	5413 independent reflections
Radiation source: fine-focus sealed tube	3730 reflections with *I* > 2σ(*I*)
Graphite monochromator	*R*_int_ = 0.044
Detector resolution: 16.1049 pixels mm^-1^	θ_max_ = 26.5°, θ_min_ = 3.5°
ω scans	*h* = −12→12
Absorption correction: multi-scan (*CrysAlis RED*; Oxford Diffraction, 2010)	*k* = −13→13
*T*_min_ = 0.899, *T*_max_ = 1.000	*l* = −17→17
42990 measured reflections	

### Refinement

**Table:**

Refinement on *F*^2^	Primary atom site location: structure-invariant direct methods
Least-squares matrix: full	Secondary atom site location: difference Fourier map
*R*[*F*^2^ > 2σ(*F*^2^)] = 0.046	Hydrogen site location: inferred from neighbouring sites
*wR*(*F*^2^) = 0.118	H-atom parameters constrained
*S* = 1.05	*w* = 1/[σ^2^(*F*_o_^2^) + (0.0523*P*)^2^ + 0.1709*P*] where *P* = (*F*_o_^2^ + 2*F*_c_^2^)/3
5413 reflections	(Δ/σ)_max_ = 0.001
353 parameters	Δρ_max_ = 0.17 e Å^−^^3^
0 restraints	Δρ_min_ = −0.20 e Å^−^^3^

### Special details

**Table:**

Experimental. 1H NMR (400 MHz, CDCl_3_): δH 2.62 (dd, *J* = 2.4, 15.2 Hz, 1H), 2.78 (dd, *J* = 5.2, 15 Hz, 1H), 3.91 (s, 3H), 5.04 (br d, 1H), 6.27 (br s, 1H), 6.33–6.39(m, 4H), 6.78 (t, *J* = 8.8 Hz, 2H), 6.84 (t, *J* = 8.8 Hz, 2H), 6.93–6.99 (m, 4H), 7.07–7.11 (m, 2H), 7.19–7.25 (m, 2H), 10.17 (br s, 1H). ^13^C NMR (100 MHz, CDCl_3_): δC 33.71, 51.19, 55.19, 57.45, 97.64, 113.88, 113.95, 114.98, 115.2, 115.30, 115.51, 115.73, 115.96, 127.88, 127.96, 128.12, 128.20, 133.62, 133.64, 138.05, 138.99, 143.06, 154.10, 156.03, 156.44, 159.67, 160.37, 160.83, 162.12, 162.80, 163.27, 168.38. IR ν_max_ (KBr): 3240, 3065, 2945, 2838, 1653, 1591, 1506, 1450, 1371, 1269, 1229, 1076, 812, 771, 685 cm^-1^. *CrysAlis PRO*, Oxford Diffraction Ltd., Version 1.171.34.40 (release 27–08-2010 CrysAlis171. NET) (compiled Aug 27 2010,11:50:40) Empirical absorption correction using spherical harmonics, implemented in SCALE3 ABSPACK scaling algorithm.
Geometry. All e.s.d.'s (except the e.s.d. in the dihedral angle between two l.s. planes) are estimated using the full covariance matrix. The cell e.s.d.'s are taken into account individually in the estimation of e.s.d.'s in distances, angles and torsion angles; correlations between e.s.d.'s in cell parameters are only used when they are defined by crystal symmetry. An approximate (isotropic) treatment of cell e.s.d.'s is used for estimating e.s.d.'s involving l.s. planes.
Refinement. Refinement of *F*^2^ against ALL reflections. The weighted *R*-factor *wR* and goodness of fit *S* are based on *F*^2^, conventional *R*-factors *R* are based on *F*, with *F* set to zero for negative *F*^2^. The threshold expression of *F*^2^ > σ(*F*^2^) is used only for calculating *R*-factors(gt) *etc*. and is not relevant to the choice of reflections for refinement. *R*-factors based on *F*^2^ are statistically about twice as large as those based on *F*, and *R*- factors based on ALL data will be even larger.

### Fractional atomic coordinates and isotropic or equivalent isotropic displacement parameters (Å^2^)

**Table:**

	*x*	*y*	*z*	*U*_iso_*/*U*_eq_	
O1	0.05599 (13)	0.35453 (13)	−0.02481 (9)	0.0522 (3)	
O2	0.25451 (13)	0.51103 (13)	0.02833 (9)	0.0553 (3)	
N1	0.38897 (13)	0.47214 (13)	0.29805 (10)	0.0384 (3)	
F1	0.16292 (17)	1.01431 (15)	0.51876 (13)	0.1073 (5)	
F2	0.97011 (11)	0.61739 (14)	0.44660 (11)	0.0792 (4)	
F3	0.36636 (18)	−0.16496 (14)	0.06807 (15)	0.1252 (6)	
F4	−0.31258 (16)	−0.11300 (15)	0.23046 (12)	0.1002 (5)	
C2	0.30643 (16)	0.54303 (16)	0.24088 (12)	0.0360 (4)	
H2	0.3679	0.5755	0.2025	0.043*	
C3	0.18153 (16)	0.44415 (16)	0.15708 (12)	0.0367 (4)	
C4	0.10236 (16)	0.35791 (16)	0.18759 (12)	0.0372 (4)	
C5	0.15506 (16)	0.36802 (17)	0.30004 (12)	0.0395 (4)	
H5A	0.1036	0.2934	0.3110	0.047*	
H5B	0.1403	0.4525	0.3504	0.047*	
C6	0.31341 (16)	0.36283 (16)	0.31870 (12)	0.0361 (4)	
H6	0.3484	0.3785	0.3943	0.043*	
C7	0.15522 (17)	0.43097 (17)	0.04696 (13)	0.0401 (4)	
C8	0.2415 (3)	0.4972 (2)	−0.08105 (16)	0.0754 (7)	
H8A	0.2506	0.4070	−0.1235	0.113*	
H8B	0.3144	0.5618	−0.0846	0.113*	
H8C	0.1507	0.5135	−0.1081	0.113*	
N9	−0.01024 (14)	0.26238 (15)	0.12273 (11)	0.0476 (4)	
H9	−0.0379	0.2597	0.0589	0.057*	
C10	0.26522 (16)	0.66857 (16)	0.31793 (12)	0.0365 (4)	
C11	0.15179 (18)	0.72134 (18)	0.28316 (14)	0.0467 (4)	
H11	0.0984	0.6782	0.2131	0.056*	
C12	0.1167 (2)	0.8370 (2)	0.35088 (17)	0.0586 (5)	
H12	0.0396	0.8709	0.3271	0.070*	
C13	0.1962 (2)	0.9001 (2)	0.45242 (17)	0.0618 (5)	
C14	0.3100 (2)	0.8534 (2)	0.49011 (16)	0.0650 (6)	
H14	0.3640	0.8990	0.5598	0.078*	
C15	0.3432 (2)	0.73677 (19)	0.42233 (13)	0.0514 (5)	
H15	0.4197	0.7033	0.4475	0.062*	
C16	0.53521 (16)	0.50829 (16)	0.33293 (12)	0.0358 (4)	
C17	0.61195 (17)	0.62124 (18)	0.32556 (14)	0.0461 (4)	
H17	0.5643	0.6733	0.2947	0.055*	
C18	0.75664 (18)	0.65696 (19)	0.36303 (15)	0.0520 (5)	
H18	0.8059	0.7324	0.3575	0.062*	
C19	0.82704 (17)	0.5806 (2)	0.40835 (14)	0.0495 (4)	
C20	0.75808 (17)	0.46901 (18)	0.41701 (13)	0.0446 (4)	
H20	0.8079	0.4179	0.4476	0.053*	
C21	0.61273 (17)	0.43295 (17)	0.37959 (12)	0.0395 (4)	
H21	0.5653	0.3570	0.3855	0.047*	
C22	0.33464 (16)	0.22233 (16)	0.25164 (13)	0.0376 (4)	
C23	0.35639 (18)	0.19043 (19)	0.14950 (13)	0.0484 (4)	
H23	0.3650	0.2581	0.1223	0.058*	
C24	0.3656 (2)	0.0598 (2)	0.08718 (16)	0.0664 (6)	
H24	0.3781	0.0386	0.0180	0.080*	
C25	0.3560 (2)	−0.0368 (2)	0.1291 (2)	0.0742 (7)	
C26	0.3379 (2)	−0.0102 (2)	0.2300 (2)	0.0761 (7)	
H26	0.3334	−0.0781	0.2572	0.091*	
C27	0.3264 (2)	0.12074 (19)	0.29126 (17)	0.0576 (5)	
H27	0.3128	0.1404	0.3601	0.069*	
C28	−0.08772 (17)	0.16492 (17)	0.15122 (13)	0.0412 (4)	
C29	−0.20066 (19)	0.19542 (19)	0.19642 (14)	0.0507 (4)	
H29	−0.2260	0.2797	0.2086	0.061*	
C30	−0.2760 (2)	0.1026 (2)	0.22363 (16)	0.0609 (5)	
H30	−0.3518	0.1232	0.2547	0.073*	
C31	−0.2376 (2)	−0.0200 (2)	0.20425 (16)	0.0609 (5)	
C32	−0.1273 (2)	−0.0550 (2)	0.15877 (16)	0.0639 (6)	
H32	−0.1042	−0.1403	0.1457	0.077*	
C33	−0.0510 (2)	0.0396 (2)	0.13272 (15)	0.0544 (5)	
H33	0.0254	0.0187	0.1026	0.065*	

### Atomic displacement parameters (Å^2^)

**Table:**

	*U*^11^	*U*^22^	*U*^33^	*U*^12^	*U*^13^	*U*^23^
O1	0.0623 (8)	0.0492 (8)	0.0333 (6)	0.0029 (6)	−0.0044 (6)	0.0117 (6)
O2	0.0675 (8)	0.0600 (8)	0.0367 (7)	0.0003 (7)	0.0078 (6)	0.0224 (6)
N1	0.0352 (7)	0.0357 (8)	0.0428 (8)	0.0036 (6)	0.0012 (6)	0.0178 (6)
F1	0.1155 (11)	0.0732 (9)	0.1012 (11)	0.0462 (8)	0.0230 (9)	−0.0112 (8)
F2	0.0355 (6)	0.0991 (10)	0.1020 (10)	0.0026 (6)	0.0006 (6)	0.0478 (8)
F3	0.1320 (14)	0.0455 (8)	0.1517 (15)	0.0252 (8)	0.0197 (11)	−0.0127 (9)
F4	0.1154 (11)	0.0859 (10)	0.1022 (11)	−0.0199 (8)	0.0182 (9)	0.0544 (9)
C2	0.0386 (8)	0.0365 (9)	0.0317 (8)	0.0047 (7)	0.0035 (6)	0.0146 (7)
C3	0.0381 (8)	0.0356 (9)	0.0314 (8)	0.0067 (7)	0.0031 (6)	0.0095 (7)
C4	0.0344 (8)	0.0385 (9)	0.0332 (8)	0.0084 (7)	0.0038 (6)	0.0088 (7)
C5	0.0388 (9)	0.0425 (10)	0.0326 (8)	0.0039 (7)	0.0067 (7)	0.0109 (7)
C6	0.0394 (9)	0.0383 (9)	0.0282 (8)	0.0049 (7)	0.0036 (6)	0.0128 (7)
C7	0.0483 (10)	0.0349 (9)	0.0352 (9)	0.0116 (8)	0.0060 (7)	0.0119 (7)
C8	0.1054 (18)	0.0825 (16)	0.0432 (11)	0.0043 (13)	0.0191 (11)	0.0329 (11)
N9	0.0469 (8)	0.0512 (9)	0.0350 (7)	−0.0055 (7)	−0.0031 (6)	0.0154 (7)
C10	0.0396 (9)	0.0344 (9)	0.0349 (8)	0.0029 (7)	0.0052 (7)	0.0156 (7)
C11	0.0471 (10)	0.0413 (10)	0.0466 (10)	0.0063 (8)	0.0006 (8)	0.0165 (8)
C12	0.0543 (11)	0.0463 (11)	0.0735 (14)	0.0167 (9)	0.0100 (10)	0.0214 (10)
C13	0.0688 (13)	0.0451 (12)	0.0626 (13)	0.0172 (10)	0.0195 (11)	0.0062 (10)
C14	0.0734 (14)	0.0617 (13)	0.0410 (11)	0.0175 (11)	0.0030 (9)	0.0010 (10)
C15	0.0565 (11)	0.0529 (11)	0.0383 (10)	0.0170 (9)	0.0025 (8)	0.0118 (9)
C16	0.0376 (9)	0.0346 (9)	0.0290 (8)	0.0054 (7)	0.0034 (6)	0.0075 (7)
C17	0.0432 (10)	0.0448 (10)	0.0502 (10)	0.0057 (8)	0.0044 (8)	0.0221 (9)
C18	0.0446 (10)	0.0494 (11)	0.0591 (12)	−0.0024 (8)	0.0094 (8)	0.0221 (9)
C19	0.0317 (9)	0.0610 (12)	0.0478 (10)	0.0051 (8)	0.0030 (7)	0.0155 (9)
C20	0.0433 (10)	0.0489 (11)	0.0362 (9)	0.0138 (8)	0.0034 (7)	0.0115 (8)
C21	0.0425 (9)	0.0366 (9)	0.0344 (8)	0.0052 (7)	0.0035 (7)	0.0111 (7)
C22	0.0336 (8)	0.0351 (9)	0.0393 (9)	0.0013 (7)	0.0024 (7)	0.0129 (7)
C23	0.0493 (10)	0.0511 (11)	0.0405 (10)	0.0146 (8)	0.0052 (8)	0.0136 (8)
C24	0.0613 (13)	0.0671 (15)	0.0492 (12)	0.0206 (11)	0.0031 (9)	−0.0010 (11)
C25	0.0605 (13)	0.0387 (12)	0.0953 (19)	0.0088 (10)	0.0072 (12)	−0.0018 (12)
C26	0.0708 (15)	0.0408 (12)	0.124 (2)	0.0070 (10)	0.0310 (14)	0.0373 (14)
C27	0.0602 (12)	0.0484 (12)	0.0743 (14)	0.0096 (9)	0.0254 (10)	0.0309 (11)
C28	0.0390 (9)	0.0409 (10)	0.0343 (9)	0.0006 (7)	0.0009 (7)	0.0094 (7)
C29	0.0530 (11)	0.0451 (11)	0.0495 (10)	0.0103 (8)	0.0117 (8)	0.0128 (9)
C30	0.0555 (12)	0.0673 (14)	0.0609 (12)	0.0068 (10)	0.0203 (10)	0.0239 (11)
C31	0.0667 (13)	0.0560 (13)	0.0542 (12)	−0.0094 (10)	0.0038 (10)	0.0256 (10)
C32	0.0830 (15)	0.0419 (12)	0.0604 (13)	0.0123 (10)	0.0023 (11)	0.0188 (10)
C33	0.0550 (11)	0.0527 (12)	0.0529 (11)	0.0169 (9)	0.0105 (9)	0.0164 (9)

### Geometric parameters (Å, º)

**Table:**

O1—C7	1.2234 (19)	C14—C15	1.382 (2)
O2—C7	1.346 (2)	C14—H14	0.9300
O2—C8	1.437 (2)	C15—H15	0.9300
N1—C16	1.3902 (19)	C16—C17	1.399 (2)
N1—C6	1.4578 (19)	C16—C21	1.404 (2)
N1—C2	1.4771 (19)	C17—C18	1.379 (2)
F1—C13	1.357 (2)	C17—H17	0.9300
F2—C19	1.3652 (19)	C18—C19	1.367 (3)
F3—C25	1.359 (2)	C18—H18	0.9300
F4—C31	1.362 (2)	C19—C20	1.363 (2)
C2—C3	1.513 (2)	C20—C21	1.385 (2)
C2—C10	1.536 (2)	C20—H20	0.9300
C2—H2	0.9800	C21—H21	0.9300
C3—C4	1.367 (2)	C22—C27	1.382 (2)
C3—C7	1.441 (2)	C22—C23	1.383 (2)
C4—N9	1.346 (2)	C23—C24	1.382 (3)
C4—C5	1.499 (2)	C23—H23	0.9300
C5—C6	1.537 (2)	C24—C25	1.355 (3)
C5—H5A	0.9700	C24—H24	0.9300
C5—H5B	0.9700	C25—C26	1.361 (3)
C6—C22	1.522 (2)	C26—C27	1.387 (3)
C6—H6	0.9800	C26—H26	0.9300
C8—H8A	0.9600	C27—H27	0.9300
C8—H8B	0.9600	C28—C29	1.378 (2)
C8—H8C	0.9600	C28—C33	1.378 (2)
N9—C28	1.430 (2)	C29—C30	1.371 (3)
N9—H9	0.8600	C29—H29	0.9300
C10—C15	1.384 (2)	C30—C31	1.357 (3)
C10—C11	1.387 (2)	C30—H30	0.9300
C11—C12	1.383 (3)	C31—C32	1.365 (3)
C11—H11	0.9300	C32—C33	1.380 (3)
C12—C13	1.355 (3)	C32—H32	0.9300
C12—H12	0.9300	C33—H33	0.9300
C13—C14	1.365 (3)		
			
C7—O2—C8	116.47 (15)	C14—C15—H15	119.2
C16—N1—C6	119.96 (12)	C10—C15—H15	119.2
C16—N1—C2	121.58 (13)	N1—C16—C17	122.49 (14)
C6—N1—C2	118.45 (12)	N1—C16—C21	120.70 (14)
N1—C2—C3	110.16 (12)	C17—C16—C21	116.81 (14)
N1—C2—C10	112.28 (12)	C18—C17—C16	121.44 (16)
C3—C2—C10	113.35 (13)	C18—C17—H17	119.3
N1—C2—H2	106.9	C16—C17—H17	119.3
C3—C2—H2	106.9	C19—C18—C17	119.45 (17)
C10—C2—H2	106.9	C19—C18—H18	120.3
C4—C3—C7	120.82 (14)	C17—C18—H18	120.3
C4—C3—C2	117.19 (13)	C20—C19—F2	119.03 (16)
C7—C3—C2	121.68 (14)	C20—C19—C18	121.73 (16)
N9—C4—C3	124.80 (14)	F2—C19—C18	119.24 (17)
N9—C4—C5	119.74 (14)	C19—C20—C21	118.97 (16)
C3—C4—C5	115.31 (13)	C19—C20—H20	120.5
C4—C5—C6	108.80 (12)	C21—C20—H20	120.5
C4—C5—H5A	109.9	C20—C21—C16	121.61 (16)
C6—C5—H5A	109.9	C20—C21—H21	119.2
C4—C5—H5B	109.9	C16—C21—H21	119.2
C6—C5—H5B	109.9	C27—C22—C23	118.21 (17)
H5A—C5—H5B	108.3	C27—C22—C6	119.57 (15)
N1—C6—C22	113.71 (12)	C23—C22—C6	122.17 (15)
N1—C6—C5	109.48 (12)	C22—C23—C24	121.17 (19)
C22—C6—C5	109.88 (12)	C22—C23—H23	119.4
N1—C6—H6	107.9	C24—C23—H23	119.4
C22—C6—H6	107.9	C25—C24—C23	118.6 (2)
C5—C6—H6	107.9	C25—C24—H24	120.7
O1—C7—O2	121.68 (15)	C23—C24—H24	120.7
O1—C7—C3	125.38 (16)	C24—C25—F3	118.5 (3)
O2—C7—C3	112.93 (14)	C24—C25—C26	122.6 (2)
O2—C8—H8A	109.5	F3—C25—C26	118.9 (2)
O2—C8—H8B	109.5	C25—C26—C27	118.4 (2)
H8A—C8—H8B	109.5	C25—C26—H26	120.8
O2—C8—H8C	109.5	C27—C26—H26	120.8
H8A—C8—H8C	109.5	C22—C27—C26	121.0 (2)
H8B—C8—H8C	109.5	C22—C27—H27	119.5
C4—N9—C28	125.43 (14)	C26—C27—H27	119.5
C4—N9—H9	117.3	C29—C28—C33	119.57 (17)
C28—N9—H9	117.3	C29—C28—N9	120.08 (16)
C15—C10—C11	117.64 (15)	C33—C28—N9	120.34 (16)
C15—C10—C2	121.57 (14)	C30—C29—C28	120.60 (18)
C11—C10—C2	120.71 (14)	C30—C29—H29	119.7
C12—C11—C10	121.19 (16)	C28—C29—H29	119.7
C12—C11—H11	119.4	C31—C30—C29	118.48 (19)
C10—C11—H11	119.4	C31—C30—H30	120.8
C13—C12—C11	118.93 (18)	C29—C30—H30	120.8
C13—C12—H12	120.5	C30—C31—F4	118.8 (2)
C11—C12—H12	120.5	C30—C31—C32	122.91 (19)
F1—C13—C12	119.10 (19)	F4—C31—C32	118.3 (2)
F1—C13—C14	118.73 (19)	C31—C32—C33	118.21 (19)
C12—C13—C14	122.17 (18)	C31—C32—H32	120.9
C13—C14—C15	118.48 (18)	C33—C32—H32	120.9
C13—C14—H14	120.8	C28—C33—C32	120.22 (18)
C15—C14—H14	120.8	C28—C33—H33	119.9
C14—C15—C10	121.58 (17)	C32—C33—H33	119.9
			
C16—N1—C2—C3	144.95 (14)	C2—C10—C15—C14	176.90 (17)
C6—N1—C2—C3	−35.70 (18)	C6—N1—C16—C17	−173.09 (14)
C16—N1—C2—C10	−87.70 (17)	C2—N1—C16—C17	6.3 (2)
C6—N1—C2—C10	91.65 (16)	C6—N1—C16—C21	5.7 (2)
N1—C2—C3—C4	46.48 (19)	C2—N1—C16—C21	−174.97 (14)
C10—C2—C3—C4	−80.28 (17)	N1—C16—C17—C18	178.43 (16)
N1—C2—C3—C7	−127.14 (15)	C21—C16—C17—C18	−0.4 (2)
C10—C2—C3—C7	106.10 (16)	C16—C17—C18—C19	0.0 (3)
C7—C3—C4—N9	−5.0 (2)	C17—C18—C19—C20	0.4 (3)
C2—C3—C4—N9	−178.70 (14)	C17—C18—C19—F2	−179.16 (16)
C7—C3—C4—C5	170.58 (14)	F2—C19—C20—C21	179.08 (15)
C2—C3—C4—C5	−3.1 (2)	C18—C19—C20—C21	−0.5 (3)
N9—C4—C5—C6	126.42 (15)	C19—C20—C21—C16	0.1 (2)
C3—C4—C5—C6	−49.43 (19)	N1—C16—C21—C20	−178.54 (14)
C16—N1—C6—C22	−71.66 (17)	C17—C16—C21—C20	0.3 (2)
C2—N1—C6—C22	108.98 (15)	N1—C6—C22—C27	151.32 (15)
C16—N1—C6—C5	165.02 (13)	C5—C6—C22—C27	−85.58 (18)
C2—N1—C6—C5	−14.34 (17)	N1—C6—C22—C23	−31.4 (2)
C4—C5—C6—N1	57.41 (16)	C5—C6—C22—C23	91.67 (17)
C4—C5—C6—C22	−68.15 (16)	C27—C22—C23—C24	1.9 (3)
C8—O2—C7—O1	−2.9 (2)	C6—C22—C23—C24	−175.43 (16)
C8—O2—C7—C3	175.84 (16)	C22—C23—C24—C25	−1.5 (3)
C4—C3—C7—O1	7.4 (3)	C23—C24—C25—F3	−179.41 (17)
C2—C3—C7—O1	−179.21 (15)	C23—C24—C25—C26	−0.1 (3)
C4—C3—C7—O2	−171.29 (14)	C24—C25—C26—C27	1.2 (3)
C2—C3—C7—O2	2.1 (2)	F3—C25—C26—C27	−179.50 (19)
C3—C4—N9—C28	175.11 (15)	C23—C22—C27—C26	−0.7 (3)
C5—C4—N9—C28	−0.3 (2)	C6—C22—C27—C26	176.63 (17)
N1—C2—C10—C15	25.0 (2)	C25—C26—C27—C22	−0.7 (3)
C3—C2—C10—C15	150.68 (16)	C4—N9—C28—C29	89.3 (2)
N1—C2—C10—C11	−158.12 (14)	C4—N9—C28—C33	−91.4 (2)
C3—C2—C10—C11	−32.5 (2)	C33—C28—C29—C30	0.4 (3)
C15—C10—C11—C12	−0.9 (3)	N9—C28—C29—C30	179.73 (16)
C2—C10—C11—C12	−177.81 (16)	C28—C29—C30—C31	−0.5 (3)
C10—C11—C12—C13	0.9 (3)	C29—C30—C31—F4	−179.58 (17)
C11—C12—C13—F1	179.37 (18)	C29—C30—C31—C32	−0.1 (3)
C11—C12—C13—C14	0.0 (3)	C30—C31—C32—C33	0.8 (3)
F1—C13—C14—C15	179.77 (19)	F4—C31—C32—C33	−179.70 (17)
C12—C13—C14—C15	−0.9 (3)	C29—C28—C33—C32	0.3 (3)
C13—C14—C15—C10	0.9 (3)	N9—C28—C33—C32	−179.00 (16)
C11—C10—C15—C14	0.0 (3)	C31—C32—C33—C28	−0.9 (3)

### Hydrogen-bond geometry (Å, º)

**Table:**

*D*—H···*A*	*D*—H	H···*A*	*D*···*A*	*D*—H···*A*
N9—H9···O1	0.86	2.05	2.695 (2)	131
C20—H20···F2^i^	0.93	2.54	3.384 (2)	152
C32—H32···O1^ii^	0.93	2.47	3.311 (3)	151

Symmetry codes: (i) −*x*+2, −*y*+1, −*z*+1; (ii) −*x*, −*y*, −*z*.
